# Does the video head impulse test replace caloric testing in the assessment of patients with chronic dizziness? A systematic review and meta-analysis

**DOI:** 10.1016/j.bjorl.2021.01.002

**Published:** 2021-02-13

**Authors:** Maria Gabriela Bonilha Vallim, Guilherme Paiva Gabriel, Raquel Mezzalira, Guita Stoler, Carlos Takahiro Chone

**Affiliations:** Universidade Estadual de Campinas (UNICAMP), Departamento de Otorrinolaringologia, Campinas, SP, Brazil

**Keywords:** Caloric test, Video head impulse test, Vestibulo-ocular reflex, vertigo

## Abstract

**Introduction:**

Caloric testing is the most frequently used test to assess peripheral vestibular function since the beginning of the 20th century. However, the video head impulse test, vHIT, has gained prominence in the field of neurotology, as it is a faster examination, easier to perform and less uncomfortable for the patient.

**Objective:**

To compare, through systematic review and meta-analysis, the proportion of altered cases between vHIT tests and caloric testing in patients with chronic dizziness, in addition to assessing the sensitivity and specificity of vHIT, with caloric testing as the gold standard.

**Methods:**

The literature search was carried out in the PubMed, Scopus, BVS-Bireme, Web of Science, Embase, Cochrane and ProQuest indexed databases, with no restrictions regarding the publication period. All articles that contained the results of the two tests were included in the evaluation of patients with dizziness. Two researchers independently conducted data selection and extraction from the studies, strictly following the inclusion and exclusion criteria defined in the research protocol. In case of disagreement during the selection, a discussion was carried out with a third evaluator.

**Results:**

Eleven of the 1293 initial articles met the eligibility criteria and were analyzed. 2670 patients were evaluated, of which 1112 (41.6%) were males and 1558 (58.4%) females, with a mean age of 51.6 years. The proportion of altered results in the vHIT was 21% (95%CI 9% –33%), and 55% in the caloric testing (95%CI 43% –67%).

**Conclusion:**

The vHIT does not substitute for caloric testing. The tests are complementary in assessing the patient with dizziness, as they describe the tonotopy of the ampullary crest at different frequency ranges of stimulation. In chronic cases, the vHIT has a low sensitivity and high diagnostic specificity in comparison to caloric testing.

## Introduction

Dizziness and vertigo are among the most common complaints by patients and affect 15% to 42% of the general population at some point during their lives.^1^, ^2^, ^3^, ^4^, ^5^, ^6^ Dizziness of vestibular origin affects approximately 8% of the population.^7^

The vestibular system contributes to body orientation and balance through interaction with several other systems, such as the visual and somatosensory ones.^8^ Of all the vestibular connections, one of the most well-described and well-known is the vestibulo-ocular reflex (VOR). This reflex is of crucial importance, as it ensures the maintenance of a clear and sharp vision by stabilizing the image on the retina during rapid head movements.^9^

Caloric testing is considered the gold standard in the evaluation of the VOR. It has been widely used since its introduction by Róbert Bárány in the early 20th century. Despite providing a qualitative and quantitative evaluation between the two labyrinths, the caloric testing has limitations. Among these, the test is performed at a very low, non-physiological frequency of approximately 0.003 Hz and cannot detect static, compensated deficits or those in active recovery states. It is a long and relatively uncomfortable examination for patients due to symptoms of nausea and dizziness.^10^

The head impulse test (HIT) is comprised of small amplitude and high-velocity head movements around the vertical axis, while the patient looks at a fixed target.^11^ It is of crucial importance in cases of acute vertigo. When altered, it is highly suggestive of a peripheral vestibular lesion. Together with spontaneous nystagmus and skew eye deviation, it has a higher sensitivity than magnetic resonance imaging (MRI) in detecting stroke in the first 48 h of its onset.^12^ The video head impulse Test (vHIT) is the computerized version of this test and, since it is more sensitive to the detection of saccades, especially “covert saccades”,^13^ it allows greater reliability regarding the VOR measurement, as well as its recording. It also allows patient followup and is considered a fundamental tool in the physical examination of patients with acute vertigo. Due to the fact that it is a fast examination with greater comfort to the patient than the caloric testing, it has also been used in the evaluation of chronic cases of VOR dysfunction. Given this scenario, the important question is whether vHIT can replace bithermal caloric testing in the diagnostic assessment of vestibular disorders in general. Several studies have compared the vHIT and caloric testing in patients with vestibular complaints. The literature states that the vHIT does not replace caloric testing in the assessment of patients with vestibular dysfunction because they represent different aspects of VOR.^10^, ^14^, ^15^

The aim of this study is to compare, through a systematic review and meta-analysis, the proportion of altered cases between the vHIT and caloric testing in patients with chronic dizziness, in addition to assessing the sensitivity and specificity of the vHIT, considering the caloric testing as the gold standard.

## Methods

### Search strategies and data sources

This systematic review followed the recommendations of the Preferred Reporting Items for Systematic Reviews and Meta-Analyses (PRISMA) method.^16^ The literature search was carried out in the PubMed, Scopus, BVS-Bireme, Web of Science, Embase, Cochrane and ProQuest indexed databases. The gray literature was consulted through the Brazilian Digital Library of Theses and Dissertations (BDTD) and EBSCOHOST. There were no restrictions regarding the period of publication. The following descriptors were used as a search strategy: (“Caloric Tests” OR “Caloric Vestibular Test”) AND (“Head Impulse Test”; OR “Video Head Impulse Test”; OR “Video-head impulse test (V-HIT)”; OR “video head impulse test (vHIT)”).

A manual search was performed of the references of the selected articles. After the search, the references for each database were exported to the EndNote X8 program (https://endnote.com/) and then those same references were exported from EndNote X8 to the Rayyan QCRI program (https: //rayyan.qcri.org/). The objectives of these two programs were to record all articles in duplicate found in the scientific literature, promote greater reliability in article selection and to proceed to the eligibility stage.

### Eligibility criteria for study selection

The eligibility criteria were defined through the PICO (Patient, Intervention, Comparison, Outcome) strategy. Therefore, adult patients (18 years or older) with dizziness or vertigo were adopted as the target population. The vHIT was considered as the intervention and the caloric testing performed in each individual as the comparison. The results had to contain a consistent and comparative analysis between the two vestibular tests that should have been compulsorily carried out on the same day or in up to 3 consecutive days, in patients with chronic dizziness (period greater than 3 months). The exclusion criteria included: 1) animal and *in vitro* experimental studies, simple reviews, abstracts, case reports, letters to the editor or book chapters; 2) language other than Portuguese, English or Spanish; 3) insufficient information for the analysis and 4) inadequate outcome.

**Figure 1:**
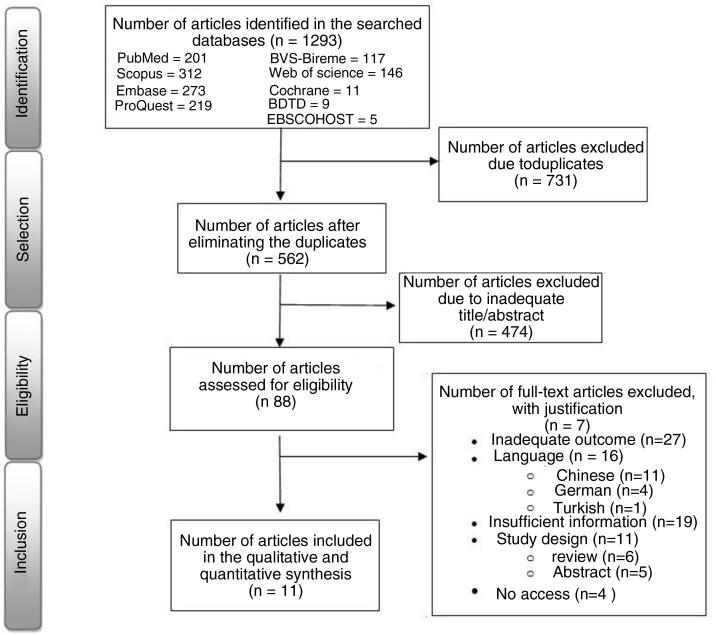
Flowchart of article selection.

Initially, all titles and abstracts were independently read by two researchers. Afterwards, according to the eligibility criteria, the pre-selected articles were read in full, adding justifications to the excluded ones. In case of disagreement in the selection, a discussion was carried out, held with an expert in neurotology as a third evaluator. The study selection flowchart is summarized in Fig. 1.

### Data extraction

The information was extracted following a standardized method, and consisted of authors' names, year of publication, study design, sample size, mean age, gender, proportion of changes in the vHIT and caloric testing and studied disease. Data on vHIT sensitivity and specificity in relation to caloric testing were also collected, when present in the studies.

### Methodological quality assessment

The assessment of quality and risk of bias of the analyzed studies was performed using the checklist of the Agency for Health Care Research and Quality (AHRQ).^17^ This checklist contains 11 evaluation criteria such as: source of information, inclusion and exclusion criteria, time period, consecutive patients, masking, quality assurance, explanation of exclusions, control of confounding factors, removal of incomplete data, complete data collection and follow-up. One item is scored as 1, if included in the article, and 0, if it is not. A score of 8 or more indicates a high-quality study (Table 1).

**Table 1:** Quality control of selected studies according to the Agency for Health Care Research and Quality (AHRQ) criteria.

Articles	Article quality according to AHRQ											
	A	B	C	D	E	F	G	H	I	J	K	Points
Blödow A et al.^18^	1	1	1	1	0	1	1	1	0	1	0	8
Blödow A et al.^19^	1	1	0	1	0	1	1	1	1	1	0	8
Burston A et al.^20^	1	0	1	1	0	1	1	1	1	1	1	9
Hannigan et al.^21^	1	0	1	1	1	1	0	1	1	1	0	8
Limviriyakul S et al.^22^	1	1	1	1	0	1	1	1	0	1	0	8
Mekki S et al.^23^	1	1	1	1	0	1	1	1	0	1	0	8
Mezzalira R et al.^24^	1	1	1	1	0	1	0	1	1	1	0	8
Oliveira LNR et al.^25^	1	1	1	1	0	1	1	1	1	1	0	9
Rambold HA et al.^26^	1	1	1	1	0	1	1	1	1	1	0	9
Rubin F et al.^27^	1	1	1	1	0	1	1	1	1	1	0	9
van Esch BF et al.^28^	1	1	0	1	0	1	1	1	1	1	0	8

A, Source of information; B, Inclusion and exclusion criteria; C, Time period; D, Consecutive patients; E, Masking; F, Quality assurance; G, Explanation of exclusions; H, Control of confounding factors; I, Withdrawal of incomplete data; J, Data integrity; K, Follow-up; 1, Present; 0, Not present or unclear.

### Statistical analysis

The software R version 3.6.0. Copyright (C) 2019 (R Foundation for Statistical Computing) was used to perform the meta-analysis. To estimate the proportions of positives for each test, random models using the Restricted Maximum Likelihood Method (REML) were utilized.

The heterogeneity analyses between the studies were performed using the Q test, and the heterogeneity quantification was performed using I^2^ statistics. This statistic estimates the proportion of heterogeneity observed in the studies, ranging from 0% to 100%, where 0% means that all heterogeneity is caused by sampling error and 100% means that all heterogeneity is due to differences between the studies.

To compare the tests, a random-effect model was adjusted for each test and a fixed-effect model to combine them. The heterogeneity in each test was assumed to be the same and Wald’s test was used to compare them. The verification of the presence of an “outlier” was carried out with the externally studentized residuals. The leave-one-out technique was used to detect influential studies. The level of significance adopted was 5% (*p* <  0.05).

## Results

Eleven studies were evaluated, which were published between 2014 and 2020, with 2670 patients, of which 1112 (41.6%) were males and 1558 (58.4%) females, with a mean age of 51.6 years. All studies compared the two tests (vHIT and caloric testing) in chronic vestibular disorders in general, where seven of them performed this analysis in specific diseases, such as Ménière’s disease, vestibular migraine, vestibular schwannoma and vestibular neuritis in the chronic phase (Table 2). The parameters used to perform both tests are listed in Table 3.

**Table 2:** Assessed characteristics of selected studies.

Authors	Year	Study	Patients (M/F)	Age	Altered vHIT	Altered CT	Disease
Blödow A et al.	2014	–	53 (17/36)	50	13 (25%)	25 (47%)	Ménière and migraine
Blödow A et al.	2015	R	69 (30/39)	58.1	30 (44%)	50 (72%)	Schwannoma
Burston A et al.	2018	P	173 (76/97)	52	26 (15%)	52 (30.1%)	Chronic vestibulopathies
Hannigan et al.	2019	R	644^a^ (213/431)	57	70 (11.5%)	90 (14.8%)	Ménière
Limviriyakul S et al.	2020	–	51 (13/38)	54.9	24 (47.1%)	39 (76.5%)	Ménière
Mekki S et al.	2020	CC	48 (27/21)	35.5	7 (29.1%)	18 (75%)	Chronic phase of VN
Mezzalira R et al.	2017	C	157 (69/88)	49	41 (26.1%)	113 (71.9%)	Chronic vestibulopathies
Oliveira LNR et al.	2019	CC	51^b^ (15/36)	45.2	5 (12.8%)	22 (56.4%)	Ménière
Rambold HÁ et al.	2015	R	1063 (510/553)	57	190 (17.9%)	397 (37.4%)	Chronic vestibulopathies
Rubin F et al.	2018	P	37 (13/24)	56	0 (0%)	34 (92%)	Ménière
van Esch BF et al.	2016	P	324 (129/195)	53	39 (12%)	113 (35%35 %)	Chronic vestibulopathies

M, Male; F, Female; R, Retrospective; P, Prospective; CC, Case-Control; C, Cross-sectional; CT, caloric testing; -, Not informed, VN, Vestibular Neuritis.

a Of the 644 patients, 606 underwent both tests.

b Of the 51 patients, 39 had symptomatic Ménière disease and were the target of statistical analysis.

**Table 3:** Parameters used to perform the vHIT and caloric testing in the analyzed studies.

Authors	Year	Video Head Impulse Test (vHIT)			Caloric testing	
		Stimulus used	Alteration criterium		Stimulus used	Alteration criterium
			Gain	Saccade		
Blödow A et al.	2014	> 10 head impulses, at 15°–20°, D 150–200 ms, PV 200°/s	Gain < 0.79	Presence of refixation saccades	Water (30°/44 °C)	LP > 25%
Blödow A et al.	2015	> 10 head impulses, at 15°–20°, D 150–200 ms, PV 200°/s	Gain < 0.79 e RA > 8.5%	Presence of refixation saccades (covert or overt)	Water (30°/44 °C)	LP > 25%
Burston A et al.	2018	> 10 head impulses; PV 150–300°/s	Gain < 0.79 at 80 ms and < 0.75 at 60ms	Presence of refixation saccades (covert or overt)	Water (cold/warm)	LP > 25%
Hannigan et al.	2019	> 20 head impulses, PV 100–300°/s	Gain < 0.8	–	Water (30°/44 °C)	LP ≥ 30%
Limviriyakul S et al.	2020	> 20 head impulses, at 10°–20°, PV 150–200°/s.	Gain < 0.8	Presence of refixation saccades (covert or overt)	Air (24°/50 °C)	LP > 25%
Mekki S et al.	2020	Short, passive, and sudden head impulse	Gain < 0.8 and AR > 8%	Presence of refixation saccades (covert or overt)	Water (30°/44 °C)	LP ≥ 20% e DP > 33%
Mezzalira R et al.	2017	> 20 head impulses, PV 150°/s and MA 1.000–2.500°/s^2^	Gain < 0.8	Presence of refixation saccades (covert or overt)	Water (30°/44 °C)	LP > 20% and ASSC < 5°/s
Oliveira LNR et al.	2019	> 20 head impulses, PV 100–250°/s, at 15°–20°, MA 1.000–2.500°/s^2^	Gain < 0.8	Presence of saccades after head movement	Air (24°/50 °C)	LP > 19% and ASSC < 5°/s
Rambold HÁ et al.	2015	> 20 head impulses. at 5°–10°, MA 750–6.000°/s^2^	Gain < 0.8	Presence of refixation saccades (covert or overt)	Water (30°/44 °C)	LP > 25% and DP > 30%
Rubin F et al.	2018	> 5 head impulses, at 10°–20°, PV > 120°/s	Gain < 0.78	Presence of refixation saccades	Water (30°/44 °C)	LP > 20%
van Esch BF et al.	2016	> 20 head impulses, at 10°–20°, D 150–200 ms, PV 150°/s	Gain < 0.8	Presence of refixation saccades (covert or overt)	Water (30°/44 °C)	LP ≥ 22% and DP ≥ 28%

A, Amplitude; D, Duration; PV, Peak Velocity; MA, Mean Acceleration; AR, Asymmetry ratio; LP, Labyrinthine predominance; DP, Directional Preponderance; ASSC, angular speed of the slow component; (–) No information.

### Comparison between caloric testing and vHIT

**Figure 2:**
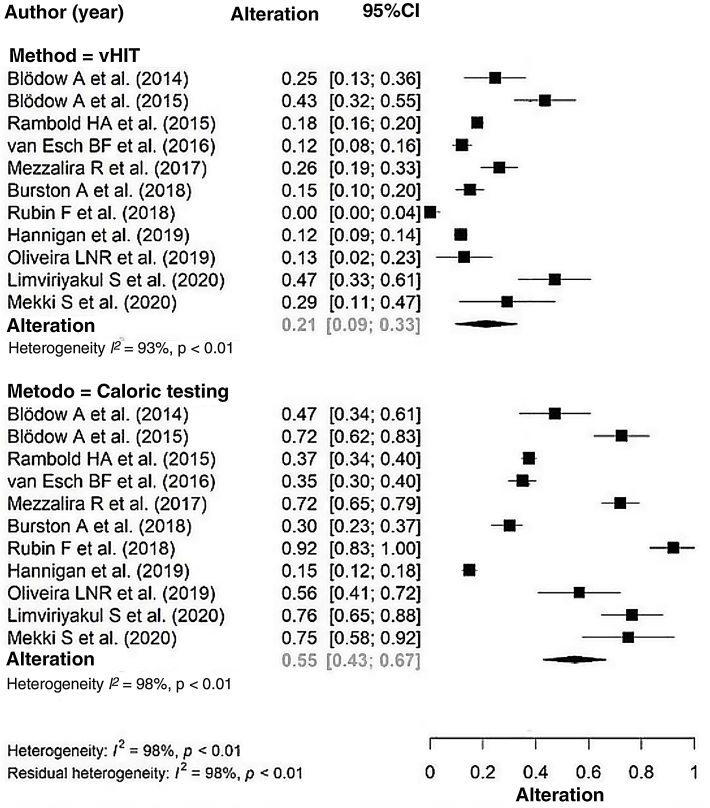
Forest plot of the percentages of the presence of alteration in each test in the analyzed studies: the probability of alteration in the caloric testing is higher than in the vHIT (0.55 × 0.21).

A comparison between the proportion of altered exams in the caloric testing and in the vHIT was performed with the 2670 patients evaluated in the eleven studies (Fig. 2). The proportion of abnormal tests in the vHIT was 21% (95% CI 9% –33%) and 55% (95% CI 43% –67%) in the caloric testing. High heterogeneity was observed between the articles, evidenced both by the Q test (*p* <  0.001) and the I^2^ measure (98%). The caloric testing showed a higher proportion of abnormal tests, with a statistically significant difference between the two exams (*p*-value < 0.001).

### VHIT sensitivity and specificity

A second meta-analysis was carried out to verify the vHIT sensitivity and specificity in relation to caloric testing, considered the gold standard test in the assessment of patients with chronic dizziness. At this stage, five articles of eleven selected ones were included, with a total of 1741 patients, since only these had complete data on true positives and negatives, and false positives and negatives, for the calculation of the assessed measures (Table 4).

**Table 4:** Variables extracted from the five included studies.

Studies	n	TP	FP	FN	TN	Sensitivity	Specificity	PPV	NPV
Burston A et al.	173	18	8	34	113	35% (23%–48%)	93% (77%–97%)	69%	77%
Mekki S et al.	24	6	1	12	5	33% (16%–56%)	83% (44%–97%)	86%	29%
Mezzalira R et al.	157	36	5	77	39	32% (24%–41%)	89% (76%–95%)	88%	33%
Rambold HA et al.	1063	142	49	256	597	36% (31%–41%)	92% (90%–94%)	74%	70%
van Esch BF et al.	324	35	4	78	207	31% (23%–40%)	98% (95%–99%)	90%	72%

TP, True Positive; FP, False Positive; FN, False Negative; TN, True Negative; PPV, Positive Predictive Value; NPV, Negative Predictive Value.

**Figure 3:**
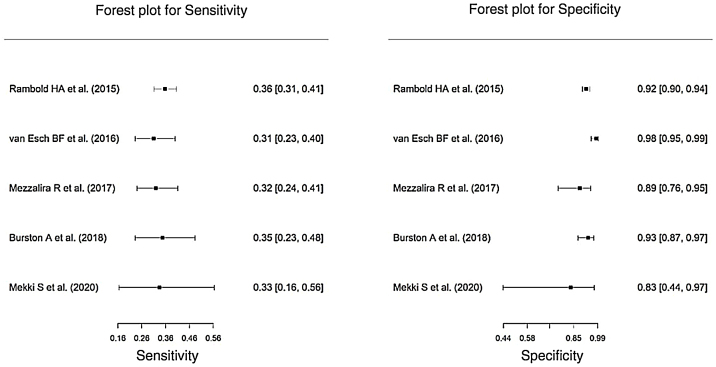
Forest plot of vHIT sensitivity and specificity in relation to caloric testing.

The vHIT sensitivity varied individually from 31% to 36% in each study, whereas the specificity varied from 83% to 98% (Fig. 3). The meta-analysis showed that in the set of selected studies, vHIT sensitivity was 0.341 (95% CI 0.307–0.377), the specificity was 0.939 (95% CI 0.896–0.965), the negative predictive value was 0.589 (95% CI 0.403 –0.752) and the positive predictive value was 0.807 (95% CI 0.712–0.876) (Table 5).

**Table 5:** vHIT efficacy in relation to Caloric Testing.

	vHIT
Sensitivity	0.3415 (0.3071–0.3776)
Specificity	0.9395 (0.8967–0.9652)
Negative Predictive Value	0.5893 (0.4039–0.7523)
Positive Predictive Value	0.8074 (0.7125–0.8764)

## Discussion

Caloric testing is the most regularly used test in clinical practice to assess vestibular function in patients with dizziness. However, it is known that the vHIT is a faster test, easier to perform and better tolerated by patients. Thus, our main objective in this systematic review and meta-analysis was to compare the two tests regarding the proportion of altered tests, in addition to assessing sensitivity and specificity of vHIT, with the caloric test as the gold standard.

Of the eleven analyzed articles, nine performed the caloric testing with water at 30 °C and 44 °C and two articles employed air at 24 °C and 50 °C. When performed at these temperatures, the stimuli with air and water are equivalent.^29^ As a criterion for caloric testing alteration, the labyrinthine predominance was found, which varied from > 19% to ≥ 30%. Some studies have also considered the directional preponderance and the angular speed of the slow component. In relation to the vHIT, all tests were performed using a standardized method, with short stimuli, with a mean amplitude of 10°-20°, peak velocity ranging between 100° –300°/s, and mean acceleration between 750–6000 m/s². The eleven studies considered the change in gain, with seven considering as altered a gain < 0.8; three studies < 0.79; and one of them < 0.78. The vast majority (90%) also considered the presence of "overt" and "covert" saccades, and two studies also calculated the ratio of asymmetry between the ears.

When comparing the two tests, we observed that the caloric testing was altered in 55% of them while the vHIT was altered in 22%, a statistically significant difference (*p-*value < 0.001). This shows that the caloric testing is more likely to show a change in vestibular function in chronic vestibular diseases. Several authors have studied this dissociation of results in the two tests. One in 6 patients with dizziness may experience disagreement between the tests.^21^, ^30^ Patients with altered caloric testing and normal vHIT frequently have peripheral vestibulopathy, while those with altered vHIT and normal caloric testing have an increased risk of central lesion.^30^

The discrepancy between the results of the two tests can be explained by the anatomophysiology of the VOR. The main VOR receptor is the ampullary crest of the labyrinth, which has specialized type I and type II cells. Type I cells are found in the center of the ampullary crest and decode the rapid head movements of high frequency, being connected to irregular afferent fibers. Type II cells, on the other hand, are found on the periphery of the ampullary crest, decode the slower head movements, with low frequency and low acceleration, and are connected to regular afferent fibers. Therefore, regular vestibular afferent fibers show a relatively greater gain at low frequencies, while irregular fibers show greater gain at high frequencies.^31^ Both vHIT and caloric testing assess unilateral VOR, but at different frequencies; the vHIT, with short, rapid head impulses, tests high frequencies (above 5 Hz), while caloric testing activates lower frequency ranges (0.003 Hz).^32^ However, the vHIT and the bithermal caloric testing differ not only in terms of frequency, but also in the type of stimulation: the vHIT, through a rapid head impulse, generates a physiological endolymphatic flow. In contrast, the caloric stimulus induces the endolymphatic flow triggered by a temperature gradient. The caloric testing stimulates the inner ear in a way that does not depend on gravity.^33^

Unilateral vestibular hypofunction is the most frequent finding in peripheral vestibular disorders, among patients with altered caloric testing. However, it is known that some central lesions also present as hypofunction in the caloric testing, as a peripheral condition. This shows that the interpretation of the dissociation between the tests must be carried out in a comprehensive context, which takes into account other associated neurological findings. The most common etiologies of peripheral vestibulopathies in the presence of an altered caloric testing and normal vHIT are Ménière’s disease and vestibular neuritis in the chronic phase.

Five studies in this systematic review specifically analyzed patients with Ménière’s disease^18^, ^21^, ^22^, ^25^^,^^27^ and the caloric testing showed more alterations than the vHIT in all of them. There were 92% of patients with abnormal caloric testing and normal vHIT in 37 patients with defined and advanced Ménière’s disease.^27^ This finding is consistent with those of other studies, similarly in defined Ménière’s disease and all of them found an abnormal caloric testing and normal vHIT.^31^, ^34^ Therefore, abnormal caloric testing associated with normal vHIT has been considered a diagnostic landmark in Meniere's disease.^27^ The dissociation in Ménière’s disease may be due to a flow of local convection of the endolymph during caloric stimulation, resulting from endolymphatic hydrops^34^ or due to the vulnerability of type II hair cells in this disease.^35^ In fact, the neural pathway involved in low frequency and low acceleration stimuli during caloric stimulation comprises type II hair cells, connected to regular afferent fibers.^36^, ^37^

The chronic phase of vestibular neuritis, another etiology frequently related with the dissociation of results in the two tests, was also included in our systematic review,^23^ which showed proportions of altered tests in the caloric testing and vHIT of 75% and 29%, respectively. Longitudinal followup studies of patients with neuritis, with analysis of tests in the acute phase and at least 1 month after the vestibular insult,^14^, ^38^ did not show any linear correlation between the vHIT and caloric testing, although both tests are shown to be altered in the acute phase. The vHIT is more likely to normalize over time. The explanation for this fact is associated with the vestibular physiology itself and the vestibular compensation process. The caloric testing stimulates the lateral semicircular canal at a very low frequency, around 0.003 Hz. In contrast, the vHIT stimulates the vestibule at a physiological frequency (0.05–5 Hz). Therefore, it is postulated that the head movements that occur in daily life can dynamically compensate for the VOR, which is measured through the vHIT, but not the caloric responses.^14^ In the presence of neuritis, or another acute vestibular dysfunction, the nerve fibers that perform high and low frequency vestibular stimulation can be independently affected and also recover at different rates, thus explaining the differences observed between the vHIT and caloric testing during these patients’ followup. As the two tests evaluate different frequencies of the semicircular canals, both are necessary for an adequate assessment.

In chronic vestibular disorders in general, our systematic review showed that the sensitivity and specificity of the vHIT in relation to caloric testing were 34% and 94%, respectively. This result is very similar to that of studies that assessed the traditional HIT (not associated with video) previously, with low sensitivity (34% –45%) and high specificity (91% –100%).^39^, ^40^ Clinically, it means that having a patient with chronic vestibular disorder, the vHIT has the capacity to achieve a correct diagnosis in only 1/3 of the cases and, therefore, it cannot be used as a replacement for caloric testing, or as a screening test. In the case of a vHIT with a normal result, a complementary assessment with the caloric testing is necessary. On the other hand, the high positive predictive value of the vHIT indicates that an altered result in this test is strongly associated with an altered caloric testing and, in this case, the latter would not be mandatory.^20^, ^21^

## Conclusion

This review shows that the vHIT is a very specific test, but little sensitive for detecting vestibular hypofunction in chronic conditions. The distinct results found between the two tests cannot be considered conflicting but rather, complementary, in the assessment of vestibular tonotopy in relation to the stimulation frequency. Therefore, the vHIT is not a replacement for caloric testing, but it constitutes a valuable assessment tool when associated with the latter.

## Conflicts of interest

The authors declare no conflicts of interest.
